# Surface-Enhanced Carboxyphenyl Diazonium Functionalized Screen-Printed Carbon Electrode for the Screening of Tuberculosis in Sputum Samples

**DOI:** 10.3390/nano12152551

**Published:** 2022-07-25

**Authors:** Muhammad Hafiznur Yunus, Nor Azah Yusof, Suhainie Ismail, Siti Suraiya Md Noor, Faruq Mohammad, Yusran Sulaiman, Nurul Hanun Ahmad Raston, Jaafar Abdullah, Ahmed A. Soleiman

**Affiliations:** 1 Institute of Nanoscience and Nanotechnology (ION2), Universiti Putra Malaysia, Serdang 43400, Selangor, Malaysia; mhn1016@yahoo.com (M.H.Y.); suhainie90@gmail.com (S.I.); yusran@upm.edu.my (Y.S.); jafar@upm.edu.my (J.A.); 2 Institute for Research in Molecular Medicine (INFORMM), Universiti Sains Malaysia, Gelugor 11800, Pulau Pinang, Malaysia; 3 Department of Chemistry, Faculty of Science, Universiti Putra Malaysia, Serdang 43400, Selangor, Malaysia; 4 School of Medical Sciences, Universiti Sains Malaysia, Kubang Kerian 16150, Kelantan, Malaysia; ssuraiya@usm.my; 5 Department of Chemistry, College of Science, King Saud University, Riyadh 11451, Saudi Arabia; 6 School of Biosciences and Biotechnology, Faculty of Science and Technology, Universiti Kebangsaan Malaysia, Bangi 43600, Selangor, Malaysia; nurulhanun@ukm.edu.my; 7 Department of Chemistry, Southern University and A&M College, Baton Rouge, LA 70813, USA; aasoleiman@aol.com

**Keywords:** tuberculosis detection, MPT64 antigen, aptamer, aptasensor, carboxyphenyl diazonium salt, sputum samples

## Abstract

Curbing tuberculosis (TB) requires a combination of good strategies, including a proper prevention measure, diagnosis, and treatment. This study proposes an improvised tuberculosis diagnosis based on an amperometry approach for the sensitive detection of MPT64 antigen in clinical samples. An MPT64 aptamer specific to the target antigen was covalently attached to the carboxyphenyl diazonium-functionalized carbon electrode via carbodiimide chemistry. The electrochemical detection assay was adapted from a sandwich assay format to trap the antigen between the immobilized aptamer and horseradish peroxidase (HRP) tagged polyclonal anti-MPT64 antibody. The amperometric current was measured from the catalytic reaction response between HRP, hydrogen peroxide, and hydroquinone, which is used as an electron mediator. From the analysis, the detection limit in the measurement buffer was 1.11 ng mL^−1^. Additionally, the developed aptasensor exhibited a linear relationship between the current signal and the MPT64 antigen-spiked serum concentration ranging from 10 to 150 ng mL^−1^ with a 1.38 ng mL^−1^ detection limit. Finally, an evaluation using the clinical sputum samples from both TB (+) and TB (−) individuals revealed a sensitivity and specificity of 88% and 100%, respectively. Based on the analysis, the developed aptasensor was found to be simple in its fabrication, sensitive, and allowed for the efficient detection and diagnosis of TB in sputum samples.

## 1. Introduction

Tuberculosis (TB) is a bacterial infection caused by *Mycobacterium tuberculosis*, with more than half of the cases progressing to pulmonary TB and a small percentage (15–20%) accounting for extrapulmonary diseases [1]. Although TB is preventable and curable, more than one million people reportedly die from it annually due to the delay in diagnosing the disease at its advanced stages [2]. TB diagnosis still relies on traditional smear microscopy and bacteriological culture, both of which have drawbacks; thus, it is critical to have updated approaches to improve and expedite the existing efforts toward eradicating this endemic.

Rapid point-of-care testing represents a good alternative for TB diagnosis, especially considering the ongoing coronavirus (COVID-19) pandemic that impairs TB diagnosis management. The World Health Organization (WHO) has reported a significant drop in the detection and reporting of TB cases, which has been impacted by the COVID-19 pandemic. This is because of the reduced healthcare capacity, movement orders, and lockdowns that restrict the ability to seek care, as well as the public stigma of TB having similar symptoms to that of COVID-19 [2]. Thus, a diagnostic test that can be performed outside the laboratory and does not require complex instruments or highly skilled professionals is urgently needed for TB confirmation.

In that view, various electrochemical detection systems with high sensitivity and specificity for TB detection have been rapidly developed due to their integrity, rapidness, and low production costs. For these electrochemical systems, the selection of a film for the surface modification of the working electrode is of utmost importance, as it has the capacity to affect the subsequent modified layers’ reliability [3]. To apply this electrode’s surface modification, a robust covalent interaction between the surface and fabricated layer is preferred over a physical adsorption approach, which is likely to leach after several washing cycles during the detection procedures. Therefore, diazonium chemistry is a widely studied technique for the covalent binding of various molecules at electrode surfaces; this is a simple and efficient technique that allows for the quick production of films with strong binding and stability, making it the preferred choice of many researchers for biosensor development [4,5,6,7]. Another vital advantage of diazonium-grafted films is the wide terminal functionality selection that links the biorecognition element to the sensor’s material surface.

Diazonium-grafted films have been utilized for the immobilization of various biomolecules, such as aptamers. Unlike antibodies, aptamers present outstanding characteristics such as greater stability, smaller size, no batch-to-batch variation, facile modification/labeling, and cost efficiency [8]. Furthermore, aptamers exhibit high binding affinity and specificity, which are crucial selection criteria for a recognition element. Therefore, incorporating the aptamers into diazonium-grafted films is expected to enhance the sensitivity and selectivity of the developed biosensor. Many previous studies have demonstrated the outstanding performance of aptamer-based biosensors in the diagnosis of TB. Thus far, those findings support the hypothesis that aptamer is an excellent biorecognition element that is very specific and capable of producing a low detection limit of down to femtomolar/g mL^−1^ in biosensors [9,10,11,12].

This study reports the use of a diazonium salt as the surface modifier and covalent linkage of the MPT64 aptamer recognition element to enhance the performance of an amperometric biosensor for TB detection. The aptamer-captured target antigen was then recognized by a secondary anti-MPT64 antibody tagged with horseradish peroxidase (HRP), forming a sandwich-format assay format. The detection signal was measured based on the enzymatic response between the peroxidase and H_2_O_2_ with the aid of hydroquinone (HQ) as an electron transfer mediator. The developed biosensor’s performance was then evaluated using clinical sputum samples from TB (+) and TB (−) individuals.

## 2. Materials and Methods

### 2.1. Reagents

From Sigma-Aldrich (St. Louis, MO, USA), 4-aminobenzoic acid (ABA), sodium nitrite (NaNO_2_), hydrochloric acid (HCl), bovine serum albumin (BSA), ethanolamine, 1-ethyl-3-(3-dimethylaminopropyl)-carbodiimide (EDC), N-hydroxysuccinimide (NHS), sodium chloride (NaCl), phosphate-buffered saline (PBS), hydroquinone (HQ), Tris-hydrochloride (Tris-HCl), 4-morpholineethanesulfonic acid (MES) potassium ferrocyanide (K_4_[Fe(CN)_6_]·3H_2_O), and human serum were purchased. Potassium ferricyanide (K_3_[Fe(CN)_6_]) and potassium chloride (KCl) were obtained from R&M Chemicals (Essex, UK). Hydrogen peroxide (H_2_O_2_, 30% *w/v*), calcium chloride (CaCl_2_), and magnesium chloride (MgCl_2_) were acquired from Merck (Darmstadt, Germany). All reagents used were of analytical grade and were used without further purification.

The buffers and solutions used in the study involve an HCl solution (1 M), MES buffer (50 mM, pH 6), PBS buffer (pH 7.4), either directly used from stock (0.1 M) or diluted to 0.01 M, bicarbonate buffer (pH 9.6), and binding buffer (20 mM Tris-HCl, 150 mM NaCl, 2 mM MgCl_2_, 2 mM KCl, and 2 mM CaCl_2_ (pH 7.4)). Ultrapure water (18.2 MΩ) was used in the preparation of all solutions.

An MPT64 aptamer was obtained from Integrated DNA Technologies (Coralville, IA, USA). The aptamer sequence was based on the study by Li et al. [13] and was further modified with an amino-modifier at the 5′ end to introduce the primary amines for binding with activated carboxylates of the diazonium layer. The sequence used in this study is 5′-AmMC6/TTTTTTTTTTGGGAGCTGATGTCGCATGGGTTTTGATCACATGA-3′. HRP-labeled streptavidin antibody was purchased from Nacalai Tesque (Kyoto, Japan). Additionally, 3,3′,5,5′-Tetramethylbenzidine (TMB) substrate was purchased from Elabscience Biotechnology (Wuhan, China). *M. tuberculosis* recombinant antigens (CFP10 and MPT64) and HRP-labeled anti-MPT64 antibodies were acquired from Cusabio (Houston, TX, USA).

### 2.2. Instrumentation

Cyclic voltammetry (CV), square wave voltammetry (SWV), and chronoamperometry measurements were performed using a μStat 8000 potentiostat controlled by the DropView 8400 Software (DropSens, Asturias, Spain). For the electrochemical impedance spectroscopy (EIS) analysis, we used an Autolab PGSTAT204 potentiostat installed with an FRA32M EIS module and the Nova version 2.1 software (Metrohm Autolab B.V., Utrecht, The Netherlands) for data acquisition. This study used a screen-printed carbon electrode (SPCE, C110D, DropSens) consisting of a carbon circular working electrode, a counter-carbon electrode, and a silver pseudo-reference electrode. A Multiskan GO spectrophotometer (Thermo Fisher Scientific, Vantaa, Finland) was used to measure the optical density during the aptamer binding study. A Fourier transform infrared (FTIR) analysis was performed using a PerkinElmer spectrometer (L1600461 Spectrum TWO DTGS, Llantrisant, UK). Contact angle analyses were run using a ThetaLite100 instrument (Biolin Scientific, Espoo, Finland). All analyses were carried out at room temperature.

### 2.3. Binding Assay of MPT64 Aptamer

The ability of an aptamer to specifically bind to the target MPT64 antigen was first determined by enzyme-linked oligonucleotide assays (ELONA). In brief, a microtiter plate (Nalgene Nunc Int., Rochester, NY, USA) was coated with 100 μL of recombinant MPT64 antigen at a 5 μg mL^−1^ concentration diluted in 0.06 M carbonate buffer (pH 9.6), and incubated overnight at 4 °C. On the next day, the plate was washed thrice with 0.01 M PBS buffer, followed by blocking with a 3% BSA solution for 1 h at 37 °C. After the washing step, 100 μL of 5 μM biotinylated MPT64 aptamer was added to the well and further incubated for 2 h at room temperature. After another cycle of washing, streptavidin-HRP conjugate at 1:10,000 was added (100 μL/well), and the plate was incubated at 37 °C for 1 h. Afterward, the plate was washed, 100 μL of TMB substrate was added and incubated for another 30 min, and the peroxidase-substrate reaction was quenched by adding 50 μL of 1 M HCl. Finally, the optical density was read at 450 nm using a Multiskan GO spectrophotometer to quantify the protein-bound aptamer-streptavidin complexes. The control experiment was performed as precisely as the same protocol, except for omitting the antigen coating part.

### 2.4. In Situ Electrografting of Carboxyphenyl Film on the Carbon Electrode and Immobilization of MPT64 Aptamer

**Figure 1:**
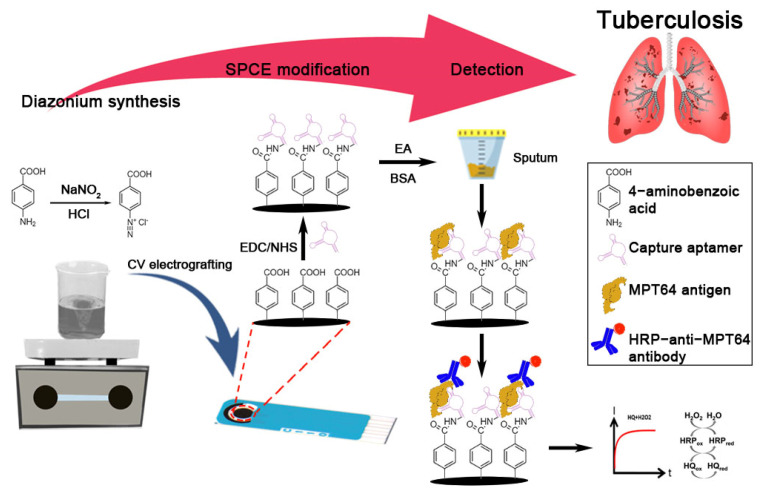
Schematic representation of fabrication of the aptasensor for the detection of MPT64 antigen.

In situ, diazonium cations were synthesized using 2 mM NaNO_2_ with 4-aminobenzoic acid in 0.5 M HCl. In situ, electrografting was performed by reducing the carboxyphenyl diazonium salt using two CV cycles from +0.5 to −0.7 V at 100 mV s^−1^. Afterwards, the electrode surface was rinsed thoroughly with deionized water to discard the loosely bound diazonium compound. The carboxyphenyl film was then activated with EDC (100 mM) and NHS (25 mM) prepared in 50 mM MES buffer (pH 6) for 1 h. After rinsing with deionized water, 10 μL of MPT64 aptamer was dropped on the carbon surface at a final concentration of 5 μM, diluted in the binding buffer, and incubated for another 90 min at room temperature. The electrode was rinsed with binding buffer before the addition of ethanolamine solution for 1 h to deactivate the remaining carboxyl active group present on the carboxyphenyl film. The modified electrode was washed with binding buffer again, and 1% of BSA was added for 1 h as a backfill process to prevent non-specific adsorption. The modified electrode was then washed with binding buffer and was ready for use or stored at 4 °C for later use. Figure 1 shows the schematic illustration of the fabrication steps of the aptasensor along with the chemistry involved in MPT64 detection.

### 2.5. Characterization of Carboxyphenyl Diazonium-Modified Electrode

The SWV was performed to characterize the blocking behavior of the aryl-grafted film on the electrode surface, and the surface characterization of the grafted diazonium layer was monitored by the contact angle measurements. A static sessile drop contact angle technique was used to measure the angle differences between the bare SPCE and after diazonium electrografting by dropping 3 μL of water onto the electrode surface. The image of the as-formed water droplet was then captured, and the contact angles were analyzed according to Young’s equation. The results are presented as an average of at least three contact angle measurements.

The stepwise assemblies were electrochemically monitored with the EIS technique measured in a 5 mM [Fe(CN)_6_]^3−/4−^ solution containing 0.1 M KCl at an amplitude of 5 mV and a frequency range from 100 kHz to 0.01 Hz since the EIS is a beneficial technique for the investigation of electron transfer kinetics and diffusion coefficients of [Fe(CN)_6_]^3−/4−^ ions on the carboxyphenyl-modified carbon electrodes. The fitting of impedance data was performed with the Nova version 2.1 software.

### 2.6. Detection Studies

The MPT64 aptamer and secondary antibody conditions were optimized to achieve the optimum parameters that produce the highest current response against the target antigen while maintaining a lower background noise simultaneously. Thus, for each optimized parameter, the amperometric signal in the presence (S) and absence (B) of the target MPT64 antigen were recorded, and the parameters that produced the largest signal-to-blank ratio (S/B) were chosen as the selection criterion.

For the electrochemical aptasensor assay, the BSA-backfilled surfaces were incubated with a range of MPT64 antigen concentrations at room temperature for 1 h. Then, the aptasensor was rinsed with binding buffer. For the selectivity study, 50 ng mL^−1^ of MPT64, the non-target *M. tuberculosis* CFP10, BSA, and human serum were incubated on the electrode surfaces. The HRP-conjugated anti-MPT64 secondary antibody was added and further incubated at room temperature for 1 h. The electrode surface was rinsed, dried, and ready for chronoamperometry measurement. The measurement was performed in 0.1 M PBS (pH 7.4) containing 2.0 mM, 0.1 mM H_2_O_2,_ and HQ, respectively, at a potential of −0.2 V (vs. Ag pseudo-reference).

### 2.7. MPT64 Antigen Detection in Spiked Serum

The spiked samples were prepared into a human serum by spiking known amounts of MPT64 antigen (ranging from 10 to 150 ng mL^−1^). A linear calibration curve was generated by plotting the analytical signal (current) vs. concentration of the spiked MPT64 antigens.

### 2.8. Clinical Sample Analysis

The ethical approval for the use of clinical samples was obtained from the Medical Research and Ethics Committee, Ministry of Health Malaysia (ethics approval no: NMRR-17-3001-39473 (IIR)), and written informed consent was obtained from the patients before sample collection. A total of 40 sputum samples were obtained, comprised of sputum from patients who were TB positive (TB (+)) confirmed by both microscopy and culture techniques (*n =* 25) and 15 non-tuberculous control samples (TB (−)). The sputum samples were diluted in the binding buffer, followed by thorough vortexing. A 5 µL of sample suspension was dropped onto the working electrode of the aptasensor and incubated for 1 h at room temperature. The rest of the steps were similar, as reported in the methodology section. All samples were collected in a sterile container, processed, and analyzed in triplicate on the same day.

### 2.9. Data Analysis

The data were analyzed statistically using GraphPad Prism version 9.2.0.332 (GraphPad Software, San Diego, CA, USA). One-way ANOVA followed by Tukey’s multiple comparison test were performed for both the aptamer binding assay and aptasensor selectivity studies. The diagnostic performance of the developed aptasensor was estimated using the receiver operating characteristic (ROC) curve analysis and then calculated the area under the curves (AUC). The optimum cut-off was derived based on the maximum value of the Youden index analysis (sensitivity + specificity − 1). The statistical differences between the TB (+) and TB (−) groups were compared using the Mann–Whitney test. All measurements were performed in triplicates, and results were reported as the mean of replicates and standard deviation (SD) from the mean value. A *p*-value < 0.05 was considered statistically significant in all the analyses.

## 3. Results

### 3.1. Principle of Aptasensor

The developed MPT64 aptasensor was adapted from a sandwich enzyme-linked format. The modified carbon electrode was rich in carboxyl functional groups that originated from carboxyphenyl diazonium-grafted film. The diazonium-grafted film serves as a base for the covalent aminated aptamer binding through the carbodiimide reaction. The aptamer acts as a biorecognition element that selectively recognizes the MPT64 antigen. The blocking steps using ethanolamine and BSA were applied to reduce the background noise from the non-specific protein binding. After each incubation step, a washing step helped remove any loosely bound reagent or protein. The sandwich format applied in this study significantly increased assay specificity since the analyte was recognized twice during the detection [14].

HRP is the most widely employed enzyme in many diagnostic techniques, mainly due to its ability to oxidize a wide range of substrates, its high availability, and its relative inexpensiveness [15,16]. Thus, this study utilizes an HRP-tagged antibody as the detection probe that reacts with the H_2_O_2_ substrate. However, since the enzyme’s reduction is usually prolonged due to slow electron transfer [17], HQ is introduced into the system to facilitate the transfer of electrons, resulting in the redox reaction between the electrode surface and the redox center of HRP [18,19]. While there is nothing new about the technique, the performance of the HRP-H_2_O_2_-HQ technique is undeniable. Many studies have proven that the technique is reliable and can improve biosensor sensitivity due to the signal amplification by the excellent enzyme catalytic reaction of HRP [20,21,22]. A remarkable electrochemical reduction signal of benzoquinone is generated from the HRP catalyzation towards the reaction between H_2_O_2_ and HQ. This signal is directly correlated with the antigens present in the samples. Therefore, the resulting reduction signal was measured using an amperometric technique for the MPT64 antigen detection in this study.

### 3.2. Binding Assay of the MPT64 Aptamer

**Figure 2:**
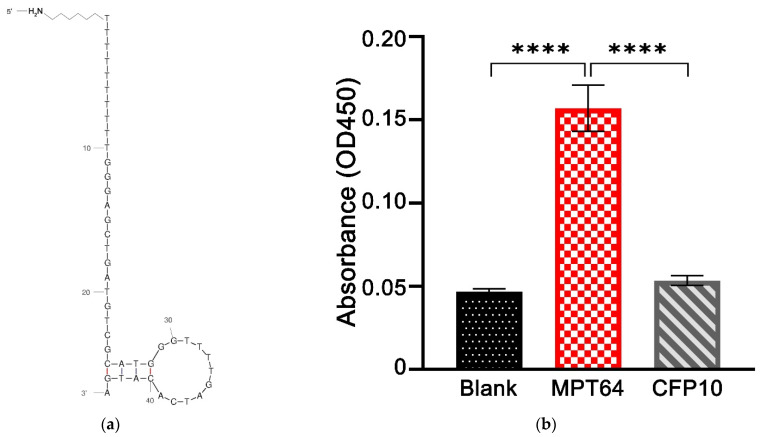
(**a**) A 2D structure of the MPT64 aptamer was predicted with the Mfold program. (**b**) The binding affinity of the MPT64 aptamer to the MPT64 target antigen. The binding ability of aptamer to MPT64 was compared with aptamer-alone control and the non-target CFP10 antigen. Statistical analysis was performed using the post hoc Tukey test. Data are presented as the mean of triplicate ± SD, **** denotes *p* < 0.0001.

The aptamer used in this study was based on previous work that showed high selectivity against MPT64 antigens [13]. Figure 2a depicts the two-dimensional structure of the MPT64 aptamer predicted with the Mfold program [23]. The binding ability of the aptamer was determined using an ELONA method by comparing the absorbance value of the MPT64 target against the aptamer-alone control and the non-target antigen-coated wells. As shown in Figure 2b, there are significant differences in the absorbance values at OD450 for the MPT64 aptamer incubated with the target antigen when compared to the aptamer-alone control (*p* < 0.0001), and the aptamer incubated with the non-target CFP10 antigen (*p* < 0.0001). Meanwhile, no significant difference was observed between the aptamer-alone control and the non-target CFP10 antigen. These results indicate that the MPT64 aptamer used in this study only recognized the binding sites specifically present in the MPT64 antigen, but not other antigens.

### 3.3. Electrografting of Diazonium Salt of 4-aminobenzoic Acid

**Figure 3:**
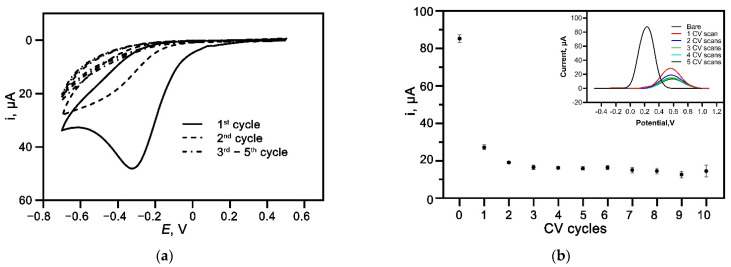
(**a**) Studies of CV for, in situ, electrografted carboxyphenyl diazonium salt generated from 2 mM 4-aminobenzoic acid and 2 mM NaNO_2_ in 0.5 M HCl at a scan rate of 100 mV s^−1^. (**b**) Distribution of the oxidation peak current for SPCE-ABA electrode at different CV cycles. Inset: SWV of bare SPCE and SPCE-ABA with different CV cycles. Measurement was performed at a scan rate, amplitude, and frequency of 125 mV s^−1^, 25 mV, and 25 Hz, respectively, in 5 mM [Fe(CN)_6_]^4^^−/3^ and 1 mM KCl.

In situ, the grafting of carboxyphenyl diazonium salt onto the electrode surface was achieved through the reduction reaction using CV scans from +0.5 to −0.7 V. A prominent irreversible cathodic peak was observed at approximately −0.35 V vs. the Ag reference electrode in the first CV scan as depicted in Figure 3a. The broad peak corresponds to the reduction in 4-aminobenzoic acid diazonium species through a one-electron transfer reaction, resulting in a 4-carboxyphenyl film covalently attached to the carbon surface. The reduced peak current significantly decreased in the second CV scan and almost disappeared in the following scans. This behavior indicates the successful attachment of aryl-diazonium salt on the carbon electrode surface that inhibits the further reduction of more diazonium cations on the surface [4,24,25]. The SWV was then performed on the diazonium-modified electrodes to study the blocking property of the grafted film. As expected, the redox activity of [Fe(CN)_6_]^3−/4−^ showed reduced intensity when increasing the number of CV reduction scans, as shown in Figure 3b inset. The blocking effect was due to the diazonium organic layers that acted as a physical barrier, preventing the [Fe(CN)_6_]^3−/4−^ redox probe from interacting with the carbon electrode surface [7,26]. Additionally, the negatively charged carboxylic group from the carboxyphenyl diazonium also contributed to the blocking behavior by repelling the [Fe(CN)_6_]^3−/4−^ anion. The plot of the SWV peak current against CV scans (Figure 3b) was then generated, showing a decrease in the SWV peak current when increasing CV scans up to two CV cycles. The subsequent CV scans showed negligible peak current changes, possibly due to the multilayers of diazonium organic layer formed on the electrode surface [4]. Thus, two CV cycles have been selected for the, in situ, electrografting procedure to prevent the formation of a multilayer that can compromise the performance of the aptasensor [27,28].

### 3.4. FTIR and Contact Angle Analyses of Carboxyphenyl Diazonium Film

**Figure 4:**
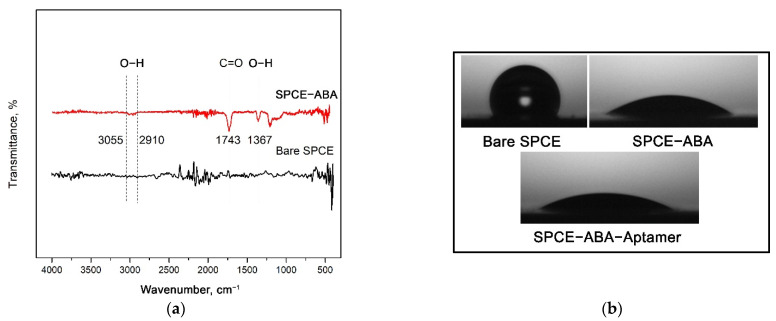
Characterizations of the diazonium-modified electrode (**a**) FTIR spectra of bare SPCE and SPCE-ABA layer. (**b**) Representative optical images for contact angle measurements on different electrode surfaces (i) bare SPCE, (ii) SPCE-ABA, and (iii) SPCE-ABA-EDC/NHS-Aptamer.

The carboxyphenyl diazonium-modified electrode was then subjected to FTIR analysis to detect the functional groups present on the electrode surface after the electrografting procedure. From the spectrum illustrated in Figure 4a, a typical peak appears at 1743 cm^−1^ ascribed to C=O, stretching the vibration of the terminal carboxylic groups. A band was observed at approximately 3055–2910 cm^−1^, corresponding to the O–H bonds found in the carboxylic acid. Another O–H band vibration was also noticed at 1367 cm^−1^, confirming the presence of the carboxylic acid functional group from the diazonium-grafted film.

In a similar way, contact angle measurements were performed to validate the binding of carboxyphenyl diazonium salt on the electrode surface. Surface hydrophilization was observed on the carbon surface after the diazonium salt was introduced to the electrode. The bare SPCE had a high contact angle value of 118.80 ± 0.6°, indicating a hydrophobic behavior that resists the wetting of water, as depicted in Figure 4b. Following this, the angle decreased significantly to 31.8 ± 1.2° after the carboxyphenyl film was grafted onto the electrode surface. The reduction in the contact angle can be explained by the formation of a strong hydrophilic carboxyl group resulting from the complete coverage of carboxyphenyl diazonium attachment on the carbon electrode surface. Further incubation with aptamer also decreased the contact angle to 25.1 ± 3.2°, possibly due to the hydrophilic phosphate bond of the DNA.

### 3.5. EIS Characterization of the Carboxyphenyl Diazonium Modified Aptasensor

**Figure 5:**
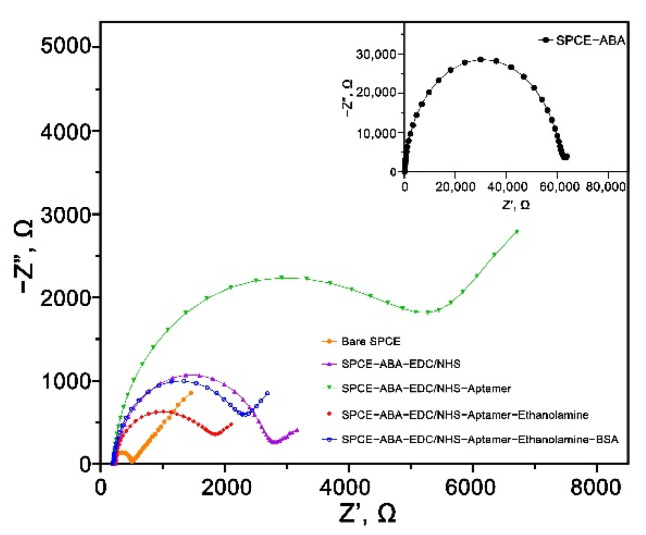
Nyquist plot of the stepwise electrode modification in 5 mM [Fe(CN)_6_]^3−/4−^ solution containing 0.1 M KCl with the frequency ranging from 100 kHz to 0.01 Hz.

EIS is a powerful electrochemical technique for investigating the interfacial properties of a modified electrode. The generated semicircle and linear lines of the Nyquist plot can provide information on the electron transfer resistance (R_ct_) and diffusion process at the electrode or electrolyte interface, respectively. Figure 5 illustrates the Nyquist plots of the stepwise modification layers of the developed aptasensor. The bare SPCE had a tiny semicircle, indicating a low R_ct_ of the non-modified carbon surface. The semicircle’s diameter increased significantly from 323.7 to 60,764 Ω, implying the successful immobilization of carboxyphenyl diazonium salt. The observed tremendous surface resistance was due to the negatively charged carboxylic group (–COOH) that limited the diffusion of [Fe(CN)_6_]^3−/4−^ redox couple to the electrode surface through electrostatic repulsion [5]. The R_ct_ then went down to 2204.2 Ω when the surface was treated with EDC/NHS, suggesting the complete activation of the -COOH group via carbodiimide reaction [29]. The impedance increased again after the immobilization of the MPT64 aptamer (R_ct_ = 4695.1 Ω) due to the repulsive effect between both the negatively charged [Fe(CN)_6_]^3−/4−^ redox couple and the nucleotide structure. Subsequently, the blocking with ethanolamine on the electrode surface showed lower surface resistance (R_ct_ = 1454.3 Ω), most likely due to the replacement of the non-specifically bound aptamers on the surface. Finally, the backfilling step with the BSA increased the R_ct_ to 1974 Ω due to the protein binding on the biosensor surface that increased its surface resistance.

### 3.6. Optimization of Aptasensor Conditions

**Figure 6:**
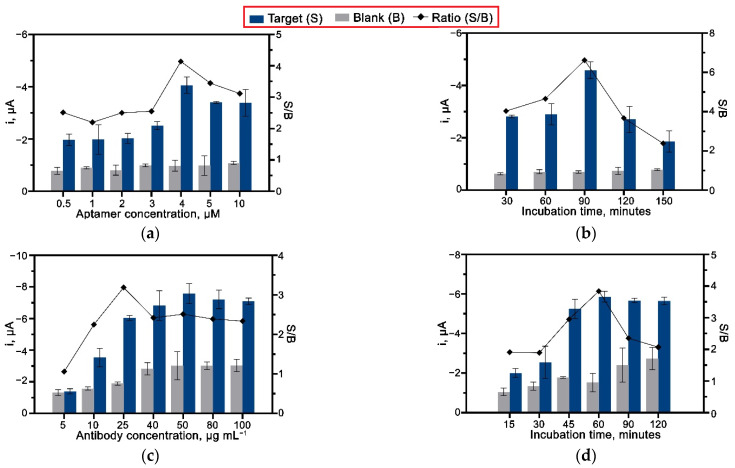
Optimization of aptasensor parameters, (**a**) concentration, and (**b**) incubation time of anti-MPT64 aptamer; (**c**) concentration and (**d**) incubation time of anti-MPT64 antibody (*n* = 3).

An optimization study was performed on both the aptamer and antibody to establish the optimal conditions for their concentration and incubation time. An amount of 25 ng mL^−1^ of MPT64 recombinant antigen was used throughout the optimization stages. Figure 6a shows the results obtained from using different aptamer concentrations ranging from 0.5 to 10 µM. The current response of the aptasensor incubated with the MPT64 antigen gradually increased with the increased amount of the aptamer concentration, reaching the highest current point with 4 µM of the aptamer loaded on the surface. Further increases in the aptamer concentration caused the drop in the current response due to the formation of a densely packed surface that restricted the aptamer binding efficiency with the target antigen [30]. The different aptamer concentrations had shown a negligible effect on the blank control electrode, and in that way, a 4 µM concentration was employed for the following studies. An immobilization period of 90 min was shown to be optimal for the complete attachment of the aptamer on the diazonium-modified surface (Figure 6b).

The loading of the HRP-conjugated anti-MPT64 antibody was optimized over the 5–100 µg mL^−1^ range value. As shown in Figure 6c, the amperometric signal increased with increased antibody concentration up to 50 µg mL^−1^. However, the results also indicated an increase in non-specific current response when high antibody concentrations (40–100 µg mL^−1^) were loaded. Accordingly, 25 µg mL^−1^ was selected as an optimal antibody concentration for the aptasensor since it produced the most significant signal-to-blank current ratio. Furthermore, an incubation time of 60 min was evidenced to be sufficient for the interaction between anti-MPT64 antibody and MPT64 antigen. The continued incubation afterward decreased the amperometric current and introduced a high background signal, as depicted in Figure 6d, due to the non-specific binding of the antibody on the electrode’s surface.

### 3.7. Analytical Performance of the Aptasensor

**Figure 7:**
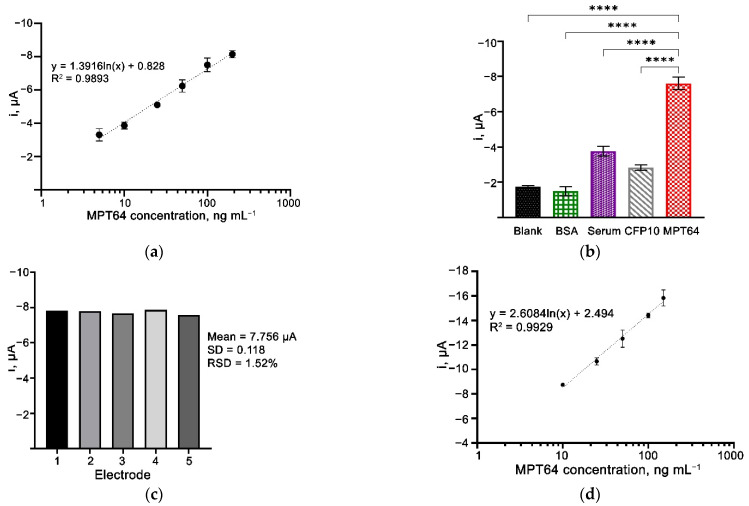
Amperometric current response of the aptasensor incubated with a series of MPT64 protein concentrations in (**a**) buffer system and (**d**) spiked serum sample. (**b**) Selectivity study of the aptasensor. Statistical analysis was performed using the post hoc Tukey test. Data are presented as the mean of triplicate ± SD, **** denotes *p* < 0.0001. (**c**) Reproducibility of the developed aptasensor incubated with 50 ng mL^−1^ of MPT64 antigen.

Under the optimized conditions, the analytical performance of the aptasensor was evaluated by plotting the calibration graph of steady-state current versus the MPT64 antigen concentration (Figure 7a). A linear relationship between the current response and the antigen was obtained in the range of 5–200 ng mL^−1^ (*R*^2^ = 0.9893). A limit of detection (LOD) of the aptasensor was derived using the 3σ/*m* formula, where σ is the SD of the blank solution and *m* refers to the slope of the calibration graph. The LOD was estimated to be 1.11 ng mL^−1^, which is comparable to the previous studies presented in Table 1. The properties of the electrodes, which include the biorecognition probe employed in the biosensor, played an essential role that affected the assay’s sensitivity. The use of aptamers has been reported to produce highly sensitive biosensors [9,30,31,32,33]. While generating a highly sensitive biosensor is always a top concern, maintaining selectivity without losing the ability to discriminate against non-target components is equally crucial. The diazonium salt property complies with the above requirements. It could passivate the residual active sites from the carbon surface and minimize the non-specific binding on the electrode surface. On the other end, diazonium produces a terminal functional group, and in this case, a carboxylic group that only attaches to the aminated-designed aptamer sequence. Thus, the grafted diazonium combined with aptamer as a biorecognition probe employed in this study can enhance the developed biosensor’s performance.

### 3.8. Selectivity and Reproducibility of the Aptasensor

An equal concentration of the MPT64 target and other non-target proteins, i.e., *M. tuberculosis* CFP10, BSA solution, and a neat human serum, were used to study the selectivity of the developed aptasensor (Figure 7). From the results, there were observed to be significant differences in the amperometric current produced by the aptasensor incubated with MPT64 as compared to the other samples having blank control, BSA, human serum, and non-target CFP10 protein (*p* < 0.0001) (Figure 7b). In the presence of MPT64, the aptamer-MPT64 antigen-HRP conjugated antibody complex was formed, allowing the catalytic reaction with substrate H_2_O_2_ and HQ to result in increased current response. These results evidenced that the developed aptasensor possessed high selectivity and could discriminate between the target MPT64 and various non-target elements. The results from our study are supported by many previous studies that enlighten the characteristics of an aptamer being very selective only toward their respective targets [5,11,39,40]. Sypabekova et al., when developing an electrochemical impedance biosensor for detecting the same MPT64 protein, found that their aptamer had excellent specificity towards MPT64 only, but not to other associated biomarkers with different diseases, i.e., human serum albumin, prostate-specific antigen, and carcinoembryonic antigen [30]. As the selection of biorecognition elements is crucial in determining an assay’s performance, the incorporation of a highly selective reagent towards its target, such as aptamer, is highly demanded.

The aptasensor’s reproducibility was evaluated by comparing the amperometric current from different electrodes (*n* = 5) prepared and tested on the same day (Figure 7c). The obtained RSD values of 1.52% indicated the reliability and reproducibility of the aptasensor during the preparation and detection steps.

### 3.9. Spiked-Sample Analysis

The present study also assessed the application of the developed aptasensor using a spiked-serum sample. MPT64 is a 25 kDa secreted protein responsible for the *M. tuberculosis* intracellular survival mechanism that resists human macrophage defense [41]. This highly antigenic secreted protein is not only present in the sputum, but there is evidence that MPT64 is also present in the serum of TB-positive patients [42]. We, thus, evaluated the potential application of the developed aptasensor using a serum sample spiked with the recombinant MPT64 protein, in addition to the patient’s sputum evaluation. A calibration curve was plotted to observe the relationship between the MPT64-spiked serum and the amperometry current response [5,29]. A concentration-dependent reaction was obtained with an increased amperometric current response from 10 ng mL^−1^ up to 150 ng mL^−^^1^ of MPT64, as demonstrated in Figure 7d. This result proved the applicability of our developed aptasensor toward the detection of MPT64 antigens in serum samples with a LOD of 1.38 ng mL^−^^1^ (*R*^2^ = 0.993).

### 3.10. Clinical Sample Analysis

**Figure 8:**
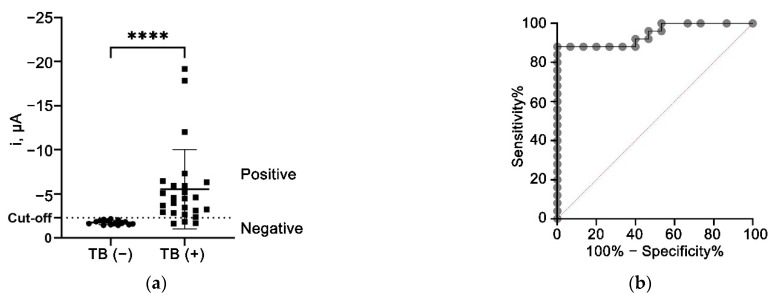
Evaluation of the aptasensor using clinical sputum samples from individuals infected with TB (TB (+)) and control non-TB individuals (TB (−)). (**a**) Scatter plot of amperometric current response between TB (+) and TB (−) individuals. Group comparison analysis was performed using the Mann–Whitney test. **** denotes *p* < 0.0001. (**b**) ROC curve analysis between TB (+) and TB (−).

The non-parametric Mann–Whitney U-test was utilized to compare the difference between the TB (+) and TB (−) groups, the results of which demonstrated that the developed aptasensor was able to differentiate between the TB (+) and TB (−) samples with a *p*-value of < 0.0001 (Figure 8a). Further analysis by ROC has also confirmed the diagnostic potential of the developed aptasensor with an AUC of 0.9440 (95% CI: 0.8757 to 1.000), as illustrated in Figure 8b. The ROC analysis also suggested −2.279 µA as the optimal cut-off value that achieved the highest diagnostic specificity while minimizing the loss of sensitivity. Based on the cut-off value, the aptasensor offered an overall diagnostic specificity and sensitivity of 100% (95 CI: 79.61–100%) and 88% (95% CI: 70.04–95.83%), respectively. The preliminary results reported in this study indicated the promising performance of the aptasensor that can discriminate between the TB (+) and TB (−) samples.

These encouraging results corroborate previous findings suggesting that the aptamer-based biosensor demonstrated excellent performance in diagnosing TB using sputum samples. Sypabekova and colleagues integrated aptamer in an interdigitated electrode platform and achieved specificity and sensitivity of 100% and 76%, respectively, when tested with sputum samples [9]. Other findings by Zhang et al. also demonstrated a good performance of aptamer-based biosensors using a multichannel series piezoelectric quartz crystal system. Their developed aptasensor targeted the whole H37Rv cells and obtained >90% sensitivity and specificity when tested with sputum samples [43]. Overall, this preliminary clinical data demonstrates an excellent and remarkable biosensor device for clinical diagnosis applications. The developed aptasensor is rapid, sensitive, and selective; thus, in accordance with the guidelines and recommendations set by the WHO, and can be employed as a rapid test for initial diagnostics to avoid unwanted delays in the treatment of TB [44].

The findings of this clinical sample analysis have to be seen in light of some limitations that future research should address. The clinical findings are solely derived from analyzing the patients’ sputum samples using the developed aptasensor and comparing the obtained results with the current gold standard method (culture technique). A limitation is that we did not conduct the quantitative analysis of the MPT64 recombinant antigen spiked in blank sputum matrix as previously performed in buffer solutions and spiked serum samples. Thus, no information on the analytical performance of the sputum sample (i.e., LOD, linearity, and selectivity) was presented that warrants further investigation in future studies. We also suggest that future studies should include the clinical evaluation of patient’s serum samples to conclude the clinical applicability of the developed aptasensor in detecting TB in human serum samples.

## 4. Conclusions

This study presents the development of aptamer-antibody sandwich-based sensing for the detection of *M. tuberculosis* MPT64 antigen. The aptamer was very selective against the MPT64 antigen, proved by the ELONA assay prior to sensor development. The aptasensor could detect down to 1.11 ng mL^−1^ and 1.38 ng mL^−1^ of antigen in the measurement buffer and spiked-serum samples, respectively. The preliminary study on clinical sputum samples concluded that the aptasensor was capable of distinguishing between the TB (+) and TB (−) samples with a diagnostic sensitivity and specificity of 88% and 100%, respectively. In summary, the proposed aptasensor could be useful in clinical sample analyses, aiming to improve current detection methods.

## Figures and Tables

**Table 1:** Comparison of the analytical performance of developed aptasensor with the previously developed electrochemical biosensor towards TB detection.

Technique	Target	Linear Range	LOD	Reference
Amperometry	CFP10	5–500 ng mL^−1^	1.22 ng mL^−1^	[5]
DPV	CFP10	20–100 ng mL^−1^	15 ng mL^−1^	[34]
DPV	CFP10-ESAT6	10–500 ng mL^−1^	1.5 ng mL^−1^	[35]
Amperometry	MPT64	0.3–50 ng mL^−1^	0.43 ng mL^−1^	[36]
SWV	Ag231	5–100 ng mL^−1^	1.0 ng mL^−1^	[37]
CV	*M. tuberculosis* DNA	0.001–100 ng µL^−1^	0.05 ng µL^−1^	[38]
Amperometry	MPT64	5–200 ng mL^−1^	1.11 ng mL^−1^	This work

LOD: Limit of detection; DPV: Differential pulse voltammetry; SWV: Square wave voltammetry, CV: Cyclic voltammetry.

## Data Availability

Not applicable.
